# Clinical significance of heparin-binding epidermal growth factor-like growth factor in peritoneal fluid of ovarian cancer

**DOI:** 10.1038/sj.bjc.6602536

**Published:** 2005-04-12

**Authors:** H Yagi, S Miyamoto, Y Tanaka, K Sonoda, H Kobayashi, T Kishikawa, R Iwamoto, E Mekada, H Nakano

**Affiliations:** 1 Department of Obstetrics and Gynecology, Graduate School of Medical Sciences, Kyushu University, 3-1-1 Maidashi, Higashi-ku, Fukuoka 812-8582, Japan; 2 Department of Obstetrics and Gynecology, Saiseikai Fukuoka General Hospital, 1-3-46 Tenjin, Chuo-ku, Fukuoka 810-0001, Japan; 3 Department of Cell Biology, Research Institute for Microbial Diseases, Osaka University, 3-1 Yamadaoka, Suita, Osaka 565-0871, Japan

**Keywords:** ovarian cancer, HB-EGF, EGFR, peritoneal fluid

## Abstract

Epidermal growth factor receptor (EGFR) has been implicated in tumour growth and extension of ovarian cancer. Peritoneal fluid in ovarian cancer patients contains various growth factors that can promote tumour growth and extension. In order to investigate the clinical significance of EGFR ligands as activating factors of ovarian cancer, we examined the cell proliferation-promoting activity and the level of EGFR ligands in peritoneal fluid obtained from 99 patients. Proliferation-promoting activity in peritoneal fluid from 63 ovarian cancer patients (OVCA) was much higher than peritoneal fluid from 18 ovarian cyst patients (OVC) and 18 normal ovary patients (NO), and the activity was suppressed only by antibodies against EGFR or heparin-binding epidermal growth factor (HB-EGF). A large difference was observed in the level of EGFR ligands between HB-EGF and TGF-*α* or amphiregulin. The concentration of HB-EGF in OVCA significantly increased compared to that in OVC or NO (*P*<0.01). No significant difference in the concentration of TGF-*α* and amphiregulin was found between the OVCA and NO or OVC groups. In peritoneal fluid, HB-EGF is sufficiently elevated to activate cancer cells even at an early stage of OVCA. These results suggested that HB-EGF in peritoneal fluid might play a key role in cell survival and in the proliferation of OVCA.

Ovarian cancer (OVCA) is the most frequent cause of cancer death among all gynaecologic cancers, and in the last 30 years current therapies have not improved cure rates (Penson *et al*, 1998). The high mortality is caused predominantly by the occult progression of the tumour into the peritoneal cavity with an initial diagnosis usually being made at an advanced stage. Tumour growth is characterised by local extension into the peritoneal cavity following the circulatory pathway of the peritoneal fluid produced by peritoneal epithelium and cancer cells. Accumulated evidence from many studies reveals that ascites from OVCA patients is a rich source of growth factor activity for OVCA cells, termed ovarian cancer activating factors (OCAFs) (Mills *et al*, 1998). The dissemination of cancer cells activated by OCAFs result in an exaggerated increase in peritoneal fluid, which in turn leads to tumour extension of OVCA.

To identify OCAFs of OVCA, various peptide growth factors and cytokines have been detected in the malignant effusions of OVCA patients (Westermann *et al*, 1997). However, the growth-promoting properties of malignant effusions *in vivo* and *in vitro* have been shown to be independent of these peptide growth factors (Westermann *et al*, 1997). Recent biochemical analysis has revealed that one possible OCAF candidate is lysophophatidic acid (LPA) (Xu *et al*, 1995; Westermann *et al*, 1998; Xiao *et al*, 2001). Lysophophatidic acid is a simple phospholipid with numerous cellular effects including growth promotion, cell cycle progression and cytoskeletal organisation (Mills and Moolenaar, 2003). However, LPA may not be the sole mediator present in ascites because the proliferation of cancer cells was lower than that induced by ascites from OVCA patients, even at optimal LPA concentrations (Xu *et al*, 1995).

Impairment of the epidermal growth factor (EGF) system has been implicated in the pathogenesis of different types of carcinomas (Salomon *et al*, 1995; Normanno *et al*, 2003). As described in the literature (Salomon *et al*, 1995; Normanno *et al*, 2003), EGF receptor (EGFR) overexpression occurs in 35–70% of all primary OVCAs and the overexpression of ErbB2 is correlated to clinical outcome. Whereas the frequency of ErbB2 overexpression is low, the frequencies of ErbB3 and ErbB4 expressions are high in OVCA. Univariate and multivariate statistical analyses have confirmed that EGFR overexpression is significantly associated with a high risk of progression in OVCA patients (Scambia *et al*, 1992). Seven ligands have been described for EGFR: EGF, transforming growth factor-*α* (TGF-*α*). heparin-binding-EGF like growth factor (HB-EGF), amphiregulin (AR), betacellulin, epiregulin, and epigen (Fischer *et al*, 2003). All are synthesised as membrane-spanning precursor molecules that have to be proteolytically processed to become fully active (Fischer *et al*, 2003). A relatively high frequency of TGF-*α* and AR has been described in ovarian carcinomas, although staining in tumours varied from weak to strong (Morishige *et al*, 1991; D'Antonio *et al*, 2002). Ovarian cancer cells are sensitive to diphtheria toxin, indicating the expression of proHB-EGF (Morimoto *et al*, 1991). We reported that HB-EGF is involved in EGFR signal transactivation induced by LPA in OVCA cell lines, and that the soluble form of HB-EGF is attributable to tumour growth on xenografted mice using OVCA cell lines (Miyamoto *et al*, 2004). On the basis of these results, it is suggested that EGFR plays a pivotal role in the acceleration and progression of ovarian caner through EGFR ligands including TGF-*α*, HB-EGF, and AR.

The concentration of EGFR ligands was examined to determine their role as tumour-promoting factors in the peritoneal fluid of OVCA patients. Peritoneal fluid of OVCA patients was also examined for proliferation-promoting and cell survival activities in OVCA cells in the absence or presence of specific inhibitory antibodies against EGFR and EGFR ligands.

## MATERIALS AND METHODS

### Patients and peritoneal fluids

All of the 99 patients in this study underwent surgery between 1994 and August 2003 at the Department of Obstetrics and Gynecology, Kyushu University Hospital. Peritoneal fluids were obtained from 99 women, who gave signed informed consent (Table 1). In all, 18 cases with normal ovaries had surgical treatment due to uterine myomas or benign gynaecologic disorders. In six cases with marked ascites and multiple disseminating sites in the peritoneum, peritoneal fluids were obtained twice, once before chemotherapy and once after three courses of chemotherapy, under a presurgical state. Peritoneal fluid supernatants were collected immediately after centrifugation (3000 **g** × 15 min), and stored at −80°C until use.

### Reagents

Recombinant human HB-EGF and synthetic LPA were purchased from R&D Systems Inc. (Minneapolis, MN, USA) and from Avanti Polar Lipids, Inc. (Alabaster, AL, USA), respectively. [^3^H]thymidine (6.7 Ci mmol^−1^) was obtained from New England Nuclear (Lachine, Quebec, Canada). Mouse anti-human EGF receptor neutralising antibody and goat anti-human HB-EGF neutralising antibody was obtained from Upstate Inc. (Lake Placid, NY, USA) and R&D Systems Inc. (Minneapolis, MN, USA), respectively. Goat anti-human TGF-*α* neutralising antibody, mouse anti-human AR neutralising antibody, mouse anti-human EGF neutralising antibody, goat anti-human epiregulin neutralising antibody and goat anti-human betacellulin neutralising antibody were also purchased from GT (Minneapolis, MN, USA). The manufacturer's instructions detail that at least 5 ng ml^−1^ of EGFR and EGFR ligands (EGF, TGF-*α*, HB-EGF, AR, betacellulin and epiregulin) can be neutralized in the use of 10 μg ml^−1^ of these antibodies, respectively. Polyclonal rabbit anti-EGFR and anti-ErbB-4 antibodies were obtained from Santa Cruz Biotechnology (Santa Cruz, CA, USA) and Upstate Biotechnology Inc. (Lake Placid, NY, USA), respectively. Peroxidase-conjugated goat anti-rabbit IgG was purchased from Zymed (San Francisco, CA, USA).

### Immunoblot

Cells were rinsed in phosphate-buffered saline (PBS) and then lysed in RIPA buffer (1% Triton X-100, 1% sodium deoxycholate, 0.1% SDS, 150 mM NaCl, 50 mM Tris (pH 8.0), 0.2 unist ml^−1^ aprotinin, 2 *μ*g ml^−1^ leupeptin, 1 *μ*g ml^−1^ pepstatin A, 2 mM phenylmethylsulphonyl fluoride). In all, 50 *μ*g of extracts wsa then subjected to SDS–polyacrylamide gel electrophoresis (SDS–PAGE) and immunoblotting analysis (Miyamoto *et al*, 2004).

### Cell proliferation-promoting assay mediated by factors in peritoneal fluid

SKOV3 cells, derived from OVCA, were maintained in RPMI-1640 (Nacalai Tesque Co. Ltd., Kyoto, Japan) supplemented with 10% (v v^−1^) fetal bovine serum (FBS). To remove extracellular matrix components, cells were detached with trypsin-EDTA, then allowed to recover for 30 min in RPMI-1640 with 10% FBS. After rinsing with serum-free medium, cells were incubated with serum-free medium at 37°C for 30 min. Cells (1 × 10^4^) were seeded on poly-lysine-coated dishes, and samples incubated with serum-free RPMI-1640 at 37°C for 1 h to assure the complete adherence of cells to the poly-lysine-coated dishes. To assess cell proliferative activity, cells were incubated in 200 *μ*l of RPMI-1640 plus 90% of each human peritoneal fluid at 37°C for 24 h; then WST-1 assay (Dojin Laboratory, Kumamoto, Japan) was performed according to the manufacturer's instructions. Further, to reconfirm the potential of the DNA polymerisation induced by peritoneal fluids, [^3^H]thymidine incorporation was examined in SKOV 3 cells, using 10% human peritoneal fluid. The cells were treated in the same manner as the WST-1 assay, and the medium was then replaced with RPMI-1640 plus 10% of each human peritoneal fluid for an additional 24 h in the absence or presence of inhibitory antibodies against EGFR, HB-EGF, TGF-*α*, amphiregulin, epiregulin, betacellulin, and EGF, or with RPMI-1640 plus 10% peritoneal fluid from patients with a normal ovary (NO) in the absence or presence of various concentrations of LPA or HB-EGF. Peritoneal fluids from 30 patients with OVCA (five cases at stage Ia, five at Ic-II, 15 at III–IV, and five of recurrence) were available for the experiment using inhibitory antibodies. [^3^H]thymidine (1 *μ*Ci well^−1^) was then added to the cell culture. After 4 h of labelling with [^3^H]thymidine, cells were washed with PBS, lysed with NaOH and treated with TCA. After adding scintillation fluid, [^3^H]thymidine uptake was measured in a *β*-scintillation counter. Each experiment was conducted in triplicate. The mean value was considered as the representative value for each experiment. The WST-1 assay and the [^3^H]thymidine incorporation were also examined in other OVCA cell lines including RMG-1 and OVMG1 cells in 10 cases with a NO and in 20 cases with OVCA.

### Inhibition of cell apoptotic assay mediated by factors in peritoneal fluid

The OVCA cell lines (1 × 10^5^) of SKOV3, RMG-1, and OVMG1 were treated in the same manner as in the WST-1 assay. Thereafter, this medium was replaced with the RPMI-1640 plus 10% of each human peritoneal fluid for an additional 24 h in the absence or presence of inhibitory antibodies against HB-EGF. Cells were harvested, pooled, and then fixed with 4% paraformaldehyde and 70% ethanol. After further washing in PBS, cells were incubated with TdT reaction reagent for 1 h at 37°C, according to the manufacturer's recommended protocol (MEBSTAIN Apoptosis Kit Direct, MBL, Co., Ltd, Japan). TUNEL-positive cells were quantified as apoptotic cells by flow cytometric analysis (Becton Dickinson, FACScan, 01-20126-xx, USA).

### Binding assay for HB-EGF

The binding of ^125^I-diphtheria toxin (DT) to HB-EGF was measured as described previously (Iwamoto *et al*, 1994). Briefly, 1 ml of peritoneal fluid was incubated with heparin-sepharose CL-6B for 5 h at 4°C. The gel was washed three times with PBS and incubated with ^125^I-DT in the presence or absence of excess unlabelled DT for 12 h at 4°C. It was then washed three times with PBS and three times with high-salt PBS. The radioactivity bound to the gel was counted with a gamma counter, and the specific binding of ^125^I-DT to the HB-EGF molecule was calculated by subtracting the radioactivity of the sample in the presence of unlabelled DT from that of the sample in the absence of unlabelled DT. The amount of HB-EGF was estimated by the standard curve obtained using recombinant human HB-EGF. All experiments were conducted in triplicate and the mean HB-EGF value was regarded as the representative value of HB-EGF in each case.

### Immunoassay for human TGF-*α* and AR

Concentrations of TGF-*α* and AR in peritoneal fluid were determined with a commercially available ELISA (Quantikine Kit, R&D Systems Inc. and ELISA Development Kit, GT, Minneapolis, MN, USA) in accordance with the manufacturer's instructions. Samples were analysed in triplicate. Levels of TGF-*α* and AR were calculated from the linear areas of the standard curves obtained using Multiskan MS, version 8.0 (Labsystems, Helsinki, Finland), respectively. The mean value was used as the representative value. The lower limits for detection of TGF-*α* and AR were 5 and 10 pg ml^−1^, respectively. When the amount was less than the detection limit, the TGF-*α* or AR value was recorded as 5 or 10 pg ml^−1^, respectively.

### Statistical analysis

The statistical significance was assessed using the Mann–Whitney test and a *P*-value less than 0.05 was considered statistically significant.

## RESULTS

**Figure 1:**
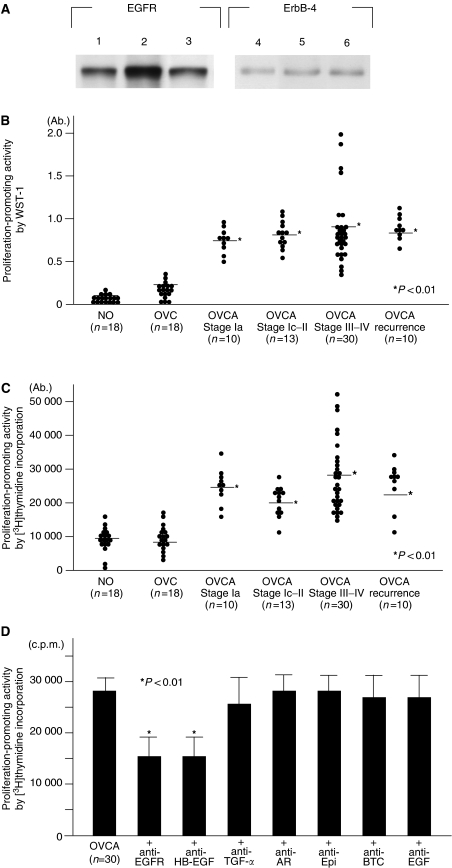
Cell proliferation activity mediated by peritoneal fluid in patients with a normal ovary (NO), an ovarian cyst (OVC), or ovarian cancer (OVCA). (**A**) Expression of EGFR and ErbB-4 protein in SKOV3, RMG-1, and OVMG1 cells. Lanes 1 and 4: SKOV3 cells. Lanes 2 and 5: RMG-1 cells. Lanes 3 and 6: OVMG1 cells. (**B**) The value of absorbance in the WST-1 assay in SKOV3 cells incubated with patients' peritoneal fluid from an NO, an OVC, and an OVCA at clinical stages Ia, Ic-II, III–IV, and recurrence. Closed circles indicate the value of absorbance in each patient. Horizontal lines indicate mean values. The *P*-value represents comparison with the levels of patients with an NO and an OVC. (**C**) The [^3^H]thymidine incorporation in SKOV3 cells incubated with patients' peritoneal fluid of a NO, an OVC, and an OVCA at clinical stages Ia, Ic-II, III–IV, and recurrence. Closed circles indicate the value of [^3^H]thymidine incorporation in each patient. Horizontal lines indicate mean values. The *P*-value represents comparison with the levels of patients with a normal ovary and an ovarian cyst. (**D**) Alterations in the [^3^H]thymidine incorporation of an ovarian cancer patient's peritoneal fluid by anti-EGFR ligand antibodies or an anti-EGFR antibody. A bar indicates the mean value and standard errors. The *P*-value represents comparison with the levels of patients in the absence of inhibitory antibodies.

To investigate the expression of EGFR and ErbB-4 in the OVCA cell lines of SKOV3, RMG-1, and OVMG1, each protein expression was examined using immunoblotting analysis. The expression of EGFR in RMG-1 cells increased remarkably compared to those in SKOV3 or OVMG1 cells. There was little difference in the expression of ErbB4 among these three lines of OVCA cells (Figure 1A).

To assess the cell proliferative property mediated by factors in peritoneal fluid, cell proliferation of SKOV3 cells was examined using the WST-1 assay after incubation with peritoneal fluid. WST-1 assay demonstrated that the value in patients with OVCA significantly increased compared to that in patients with a NO or ovarian cyst (OVC) (Figure 1B and Table 2). To address whether peritoneal fluid from OVCA patients actually stimulates cancer cell proliferation, the proliferation of SKOV3 cells was examined by [^3^H]thymidine incorporation into DNA. In the absence of the patient's peritoneal fluid, SKOV3 cells showed [^3^H]thymidine incorporation at a minimum value (5800±1200 cpm). Addition of peritoneal fluid resulted in increased DNA synthesis in all cases, but peritoneal fluid from OVCA patients enhanced DNA synthesis much highly than that from NO and OVC (each *P*<0.01) (Figure 1C and Table 2). In the WST-1 assay, the absorbances of the 10 cases with a NO and the 20 cases with OVCA were 0.31±0.12 and 0.62±0.23 (mean±standard deviation) (RMG-1 cells), and 0.30±0.13 and 0.69±0.23 (OVMG1 cells), respectively. In RMG-1 and OVMG1 cells, the proliferative activity in peritoneal fluid of OVCA patients was significantly enhanced, compared with that in NO (both *P*<0.01). In [^3^H]thymidine incorporation, the values in the 10 cases with a NO and the 20 cases with OVCA were 5823±1066 c.p.m. (count per minute) and 9241±1553 c.p.m (mean±standard deviation) (RMG-1 cells), and 6903±1134 c.p.m. and 12572±1951 c.p.m. (OVMG1 cells), respectively. In RMG-1 and OVMG1 cells, the DNA polymerisation property mediated by peritoneal fluid in OVCA was also significantly elevated, compared with that in NO (both *P*<0.01). These results indicate that peritoneal fluid in patients with OVCA possesses a significant cell proliferative property.

To examine which EGFR ligands contribute to SKOV3 cell proliferation in a patient's peritoneal fluid, the effects of peritoneal fluid on SKOV3 cell DNA synthesis were measured in the absence or presence of an inhibitory antibody against EGFR or each EGFR ligand. [^3^H]thymidine incorporation was significantly reduced in the presence of anti-EGFR or anti-HB-EGF neutralising antibody, while neutralising antibodies against TGF-*α*, AR, epiregulin, betacellulin, and EGF had no effect on [^3^H]thymidine incorporation in SKOV3 cells (Figure 1D). The contribution of epigen was not determined, as no antibodies against epigen are available. In the presence of inhibitory antibodies against HB-EGF or EGFR, the values of [^3^H]thymidine incorporation were 7301±1093 and 7308±1120 c.p.m (RMG-1 cells), and 9513±1510 and 9160±1296 c.p.m. (OVMG1 cells), respectively. [^3^H]thymidine incorporation was also significantly reduced in the presence of anti-EGFR or anti-HB-EGF neutralising antibodies using RMG-1 or OVMG1 cells.

**Figure 2:**
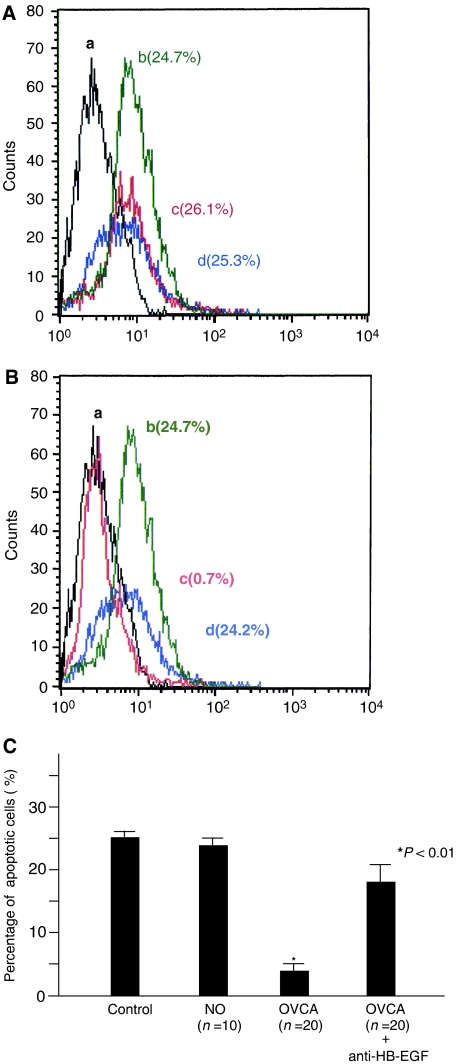
Cell survival activity mediated by peritoneal fluid in patients with a normal ovary or ovarian cancer. Flow cytometric analysis for apoptotic cells in SKOV3 cells after incubation with peritoneal fluid of a normal ovary (**A**) and ovarian cancer (**B**). Control (a: black line). Under serum-free condition (b: green line). Incubation with 10% peritoneal fluid in the absence (c: red line) or presence (d: blue line) of an inhibitory antibody against HB-EGF. Each percentage indicates the ratio of apoptotic cells in SKOV3 cells. (**C**) Alteration in the percentage of apoptotic cells after incubation with the peritoneal fluid from a normal ovary or ovarian cancer. A bar indicates the mean value and standard errors. The *P*-value represents comparison with the levels of patients with a normal ovary.

To evaluate the role of HB-EGF in cell survival in peritoneal fluid, apoptotic cells were analysed using the FACScan after staining cells by TUNEL methods in 10% human peritoneal fluid with an absence or presence of an inhibitory antibody against HB-EGF. Significant apoptotic cells in RMG-1 and OVMG1 cells were not observed even under serum-free conditions. In SKOV3 cells, 25.1±1.4% (mean±standard deviation) were detected as TUNEL-positive under serum-free conditions (Figure 2C). In a patient with a NO, there was no difference in the percentage of apoptotic cells between an absence or presence of peritoneal fluid, or between an absence or presence of the inhibitory antibody plus peritoneal fluid (Figure 2A). In a patient with OVCA, the percentage of apoptotic cells incubated with peritoneal fluid markedly decreased, compared to that incubated without peritoneal fluid (Figure 2B). As well, peritoneal fluid plus the inhibitory antibody against HB-EGF blocked any decrease of apoptotic cells (Figure 2B and C). In the 10 cases with a NO or the 20 cases with OVCA, the percentages of apoptotic cells were 24.6±4.6 or 3.3±2.2%, respectively, after incubation with peritoneal fluid (Figure 2C). In peritoneal fluid from OVCA patients in the presence of the inhibitory antibody against HB-EGF, the percentage of apoptotic cells significantly increased at 18.2±5.2%, compared with that in the absence of the inhibitory antibody (*P*<0.01) (Figure 2C). These results suggest that HB-EGF in the peritoneal fluid of OVCA patients may contribute to cell survival in OVCA cells.

**Figure 3:**
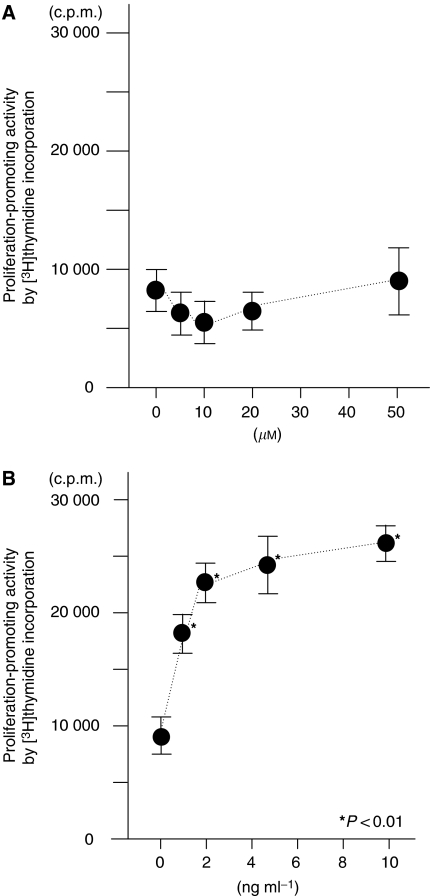
Mitogenic activity of LPA (**A**) and HB-EGF (**B**) in the presence of peritoneal fluid. Each point indicates the mean and standard deviation of [^3^H]thymidine incorporation. The *P*-value represents comparison with the levels of [^3^H]thymidine incorporation incubated in the medium containing peritoneal fluid of a patient with a normal ovary.

The present study suggests that HB-EGF levels may increase in peritoneal fluid from OVCA patients, and that HB-EGF is one of the factors in peritoneal fluid from OVCA patients that promotes tumour growth. Thus, HB-EGF, in addition to LPA, should be included as a member of OCAFs. To compare HB-EGF with LPA for proliferation-promoting activity, SKOV3 cells were cultured either with HB-EGF or LPA in a culture medium containing peritoneal fluid of NO patients. Although the concentration of LPA in the peritoneal fluid of OVCA patients is reportedly from 10 to 20 *μ*M (Xu *et al*, 1995; Westermann *et al*, 1998; Xiao *et al*, 2001), LPA (0–50 *μ*M) did not show any growth-promoting effect in the present cell system (Figure 3A). In contrast, HB-EGF enhanced SKOV3 cell proliferation in a dose-dependent manner (Figure 3B), even at concentrations of 1–10 ng ml^−1^, which are comparable to the levels of HB-EGF in peritoneal fluid of OVCA patients. These results indicate that HB-EGF induced significant cell proliferation, even in the presence of a concentration of 1 ng ml^−1^.

**Figure 4:**
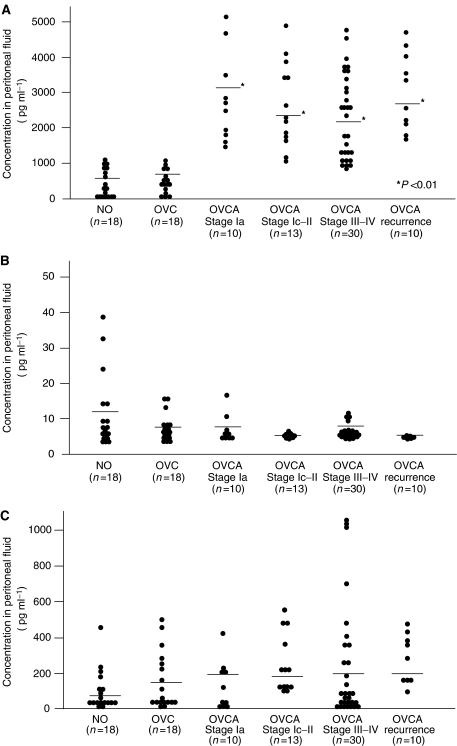
Distribution of HB-EGF (**A**), TGF-*α* (**B**), and AR (**C**) concentration in peritoneal fluid among patients with a normal ovary (NO), an ovarian cyst (OVC), or ovarian cancer (OVCA) at clinical stages Ia, Ic-II, III–IV, and recurrence. Closed circles indicate the value of EGFR ligand concentration in each patient. Horizontal lines indicate mean values. The *P*-value represents comparison with the levels of patients with an NO or an OVC.

To gain an insight into the role of EGFR ligands in peritoneal fluid of OVCA patients, the concentrations of HB-EGF, TGF-*α*, and AR were determined in the peritoneal fluid of patients with a NO , OVC, and OVCA. Heparin-binding epidermal growth factor levels were significantly enhanced in all stages in OVCA patients compared with levels in NO and OVC patients (*P*<0.01) (Figure 4A and Table 2). Transforming growth factor--*α* levels were quite low (less than 40 pg ml^−1^) in all cases, and 46 out of 99 cases (four cases of NO, six cases of OVC, 30 cases of OVCA, and six cases of recurrence of OVCA) showed levels lower than the detection limit (5 pg ml^−1^). Amphiregulin levels were scattered among the cases, but were less than 1000 pg ml^−1^ except in three OVCA cases. No significant differences were found in TGF-*α* and AR levels among the patients with NO, OVC, and OVCA (Figure 4B, C and Table 2). In addition to the significant increase of HB-EGF levels in OVCA patients, the concentration of HB-EGF in the peritoneal fluid of OVCA patients was much higher than those of TGF-*α* and AR (Figure 4), suggesting that HB-EGF is a major EGF family ligand, and is involved in tumour growth and OVCA extension.

**Figure 5:**
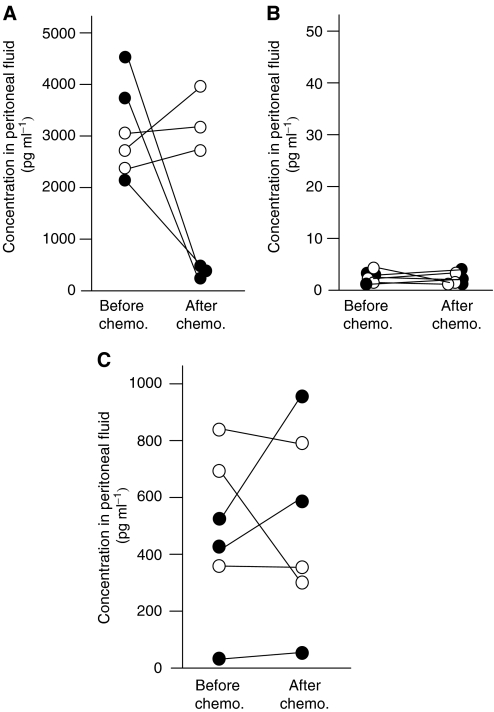
Changes in concentrations of HB-EGF (**A**), TGF-*α* (**B**), and AR (**C**) in the peritoneal fluid among patients with ovarian cancer between pre- and post-chemotherapy. Open circles indicate the concentration of each peritoneal fluid from chemotherapy-non-responding patients. Closed circles indicate the concentration of each peritoneal fluid from chemotherapy-responding patients.

To elucidate the relationship between EGFR ligands and clinical outcome, the amounts of EGFR ligands in peritoneal fluid were compared between before and after chemotherapy. Of the six cases examined, three cases showed a good response to neo-adjuvant chemotherapy and a marked reduction in tumour size and peritoneal fluid. In these cases, computed tomography indicated the disappearance of the tumour in the abdomen with six courses of chemotherapy. The other three cases did not respond to neo-adjuvant chemotherapy and there was no marked effect on tumour growth or the volume of peritoneal fluid. The levels of HB-EGF were dramatically reduced in the former cases, but were slightly increased in the latter ones (Figure 5A). No significant changes in TGF-*α* and AR were observed between pre- and post-chemotherapy (Figure 5B and C). These results suggest that HB-EGF levels in peritoneal fluid might reflect the response to chemotherapy.

## DISCUSSION

In this study, we have shown the following: (1) HB-EGF levels in peritoneal fluid were elevated at all stages and at recurrence, and levels of HB-EGF were sufficient to proliferate and to allow survival of OVCA cells, whereas the levels of the other five EGFR ligands in peritoneal fluid had little effect on the proliferation of OVCA cells. (2) Cell proliferation of OVCA cells was stimulated by the addition of HB-EGF, but not LPA, in *in vitro* study. (3) Changes in the amount of HB-EGF were reflected by therapeutic efficacy in OVCA patients. Taken together, the present study suggests that HB-EGF plays a pivotal role in tumour growth and extension of OVCA. In this study, however, cell proliferative activities in SKOV3, RMG-1, or OVMG1 cells, which were mediated by peritoneal fluid from OVCA patients, were not always dependent on the expression of EGFR or ErbB-4. It remains open to debate how EGFR or ErbB-4 is involved in the cell proliferation mediated by peritoneal fluid in OVCA patients.

Among the members of EGF family growth factors, HB-EGF, AR, and betacellulin have heparin-binding properties (Raab and Klagsbrun, 1997; Iwamoto and Mekada, 2000; Strachan *et al*, 2001). Heparin-binding epidermal growth factor is also known to have a broad spectrum of biological activities including mitogenic activity, chemotaxis, adhesion, and angiogenesis (Raab and Klagsbrun, 1997; Iwamoto and Mekada, 2000). Increasing evidence indicates that HB-EGF is involved in various pathophysiological disorders. Recently, it has been shown that EGFR is transactivated by a variety of stimuli through the ectodomain shedding of EGFR ligands, and that HB-EGF plays a central role in this process (Prenzel *et al*, 1999). Lysophophatidic acid and other ligands for G protein coupled receptors (GPCR) also transactivate EGFR through ectodomain shedding of HB-EGF or AR (Prenzel *et al*, 1999; Umata *et al*, 2001; Gschwind *et al*, 2003). In OVCA, LPA has been identified as a candidate for an OCAF (Xu *et al*, 1995; Westermann *et al*, 1998; Xiao *et al*, 2001). In our study, LPA did not stimulate SKOV3 cell proliferation in the presence of peritoneal fluid of NO patients; however, the effect of LPA might be dependent on the cell system. In contrast, HB-EGF stimulated SKOV3 cell proliferation in the same culture conditions. Therefore, it is likely that LPA is an OCAF, but it stimulates OVCA cell proliferation by inducing the ectodomain shedding of HB-EGF, rather than directly acting by itself on cancer cells.

In OVCA, LPA regulates the production of LPA itself through the activation of phospholipase D and phospholipase A2 (Eder *et al*, 2000). Similarly, HB-EGF induces HB-EGF gene expression itself by activating mitogenic properties (Tan *et al*, 1994). In addition, LPA and HB-EGF appear to influence their reciprocal production. Lysophophatidic acid can enhance the gene expression of HB-EGF (Goetzl *et al*, 1999), as well as induce the ectodomain shedding of HB-EGF (Prenzel *et al*, 1999; Umata *et al*, 2001). Heparin-binding epidermal growth factor activates EGFR, and the activated EGFR enhances the activity of phospholipase D, which in turn causes the production of LPA (Yeo and Exton, 1995). Considering this evidence as a basis, HB-EGF and LPA might collaborate in inducing cell proliferation and in amplifying their own production. In this study, we showed that HB-EGF is elevated in the ascitic fluid of OVCA patients even at an early stage. Lysophophatidic acid may contribute to the production of HB-EGF, especially in an early phase of OVCA.

Heparin-binding epidermal growth factor in peritoneal fluid is generated mainly from cancer cells and peritoneal mesothelial cells, which have the same origin as coelomic epithelium of the NO. Our study showed that HB-EGF levels were already elevated even in the ascitic fluid of patients with stage Ia OVCA, whereas OVCA cells are enclosed within the cyst wall. This suggests that HB-EGF is not secreted from cancer tissues in an early stage. In peritoneal mesothelial cells, HB-EGF is constitutively expressed and produced by cytokine stimulation (Jayne *et al*, 2000). Therefore, it is possible to speculate that during early OVCA, mainly peritoneal mesothelial cells produce HB-EGF in the peritoneal fluid by stimulation of a variety of cytokines or by LPA. In advanced stages of OVCA, however, the HB-EGF levels in the peritoneal fluid appear to be correlated with the tumour state in the peritoneal cavity, as HB-EGF levels of patients' fluids were largely reduced after chemotherapy in chemotherapy-responding cases. Therefore, in advanced OVCA, both cancer and peritoneal mesothelial cells might produce HB-EGF.

These results are the first demonstration of HB-EGF as an OCAF. Heparin-binding epidermal growth factor is known to enhance cell motility as well as cell proliferation. Therefore, elevated HB-EGF in peritoneal fluid may contribute to not only survival and proliferation of cancer cells, but also to their dissemination into the peritoneal cavity. It is easier to measure HB-EGF levels in peritoneal fluid compared with LPA, suggesting that HB-EGF is a promising bioactive marker of OVCA. For therapy, relevant LPA antagonists have yet to be developed, while Iressa, a specific EGFR tyrosine-kinase inhibitor, is not effective in OVCA (Baselga *et al*, 2002). Thus, the development of therapeutic tools against HB-EGF would allow the exploration of novel targeting therapies for OVCA.

## Figures and Tables

**Table 1:** Clinical data for patients

			**Histological types**	
**Variable**	**No. of** **patients**	**Age (years)** **(mean±s.d.)**	**Serous**	**Others**
Normal ovary	18	55.5±17.8		
Ovarian cyst	18	54.6±19.7	10	8
Ovarian cancer, stage Ia	10	57.2±14.3	7	3
Ovarian cancer, stage Ic–II	13	55.8±10.6	8	5
Ovarian cancer, stage III–IV	30	59.3±16.2	22	8
Ovarian cancer, recurrence	10	58.2±11.2	7	3

**Table 2:** Concentrations of HB-EGF, TGF-*α*, and amphiregulin, and cell proliferation properties in peritoneal fluid

	**Concentration of EGF ligands (pg ml^−1^, mean±s.d.)**			**Proliferation-promoting activities (mean±s.d.)**	
**Variable**	**HB-EGF**	**TGF-*α***	**Amphiregulin**	**[^3^H]Thymidine** **incorporation (c.p.m.)**	**WST-1 assay (abs.)**
Normal ovary (*N*=18)	483±495	11.12±13.01	98±236	9216±5777	0.12±0.11
Ovarian cyst (*N*=18)	653±616	7.37±3.67	186±322	8990±5381	0.26±0.18
Ovarian cancer, stage Ia (*N*=10)	3090±2286*	7.45±4.01	214±183	21 747±8678**	0.71±0.29***
Ovarian cancer, stage Ic–II (*N*=13)	2132±1394*	8.33±7.47	203±220	18 654±4988**	0.76±0.33***
Ovarian cancer, stage III–IV (*N*=30)	2053±1204*	5.14±0.62	225±755	24 556±10 152**	0.86±0.66***
Ovarian cancer, Recurrence (*N*=10)	2544±1098*	2.01±5.12	212±175	21 035±9755**	0.78±0.28***

* *P*<0.01 for comparison of HB-EGF level *vs* normal ovary.

** *P*<0.01 for comparison of c.p.m. count *vs* normal ovary.

*** *P*<0.01 for comparison of absorbance *vs* normal ovary.

## References

[bib1] Baselga J, Rischin D, Ranson M, Calvert H, Raymond E, Kieback DG, Kaye SB, Gianni L, Harris A, Bjork T, Aberbuch SD, Feyereislova A, Swaisland H, Rojo F, Albanell J (2002) Phase I safety, pharmacokinetic, and pharmacodynamic trial of ZD1839, a selective oral epidermal growth factor receptor tyrosine kinase inhibitor, in patients with five selected solid tumor types. J Clin Oncol 20: 4292–4302 12409327 10.1200/JCO.2002.03.100

[bib2] D'Antonio A, Losito S, Pignata S, Grassi M, Perrone F, De Luca A, Tambaro R, Bianco C, Gullick WJ, Johnson GR, Iaffaioli VR, Salomon DS, Normanno N (2002) Transforming growth factor alpha, AR and cripto-1 are frequently expressed in advanced human ovarian carcinomas. Int J Oncol 21: 941–948 12370739

[bib3] Eder AM, Sasagawa T, Mao M, Aoki J, Mills GB (2000) Constitutive and lysophosphatidic acid (LPA)-induced LPA production: role of phospholipase D and phospholipase A2. Clin Cancer Res 6: 2482–2491 10873103

[bib4] Fischer OM, Hart S, Gschwind A, Ullrich A (2003) EGFR signal transactivation in cancer cells. Biochem Soc Trans 31: 1203–1208 14641026 10.1042/bst0311203

[bib5] Goetzl EJ, Kong Y, Kenney JS (1999) Lysophospholipid enhancement of human T cell sensitivity to diphtheria toxin by increased expression of heparin-binding epidermal growth factor. Proc Assoc Am Physicians 111: 259–269 10354366 10.1046/j.1525-1381.1999.99116.x

[bib6] Gschwind A, Hart S, Fischer OM, Ullrich A (2003) TACE cleavage of proAR regulates GPCR-induced proliferation and motility of cancer cells. EMBO J 22: 2411–2421 12743035 10.1093/emboj/cdg231 PMC155989

[bib7] Iwamoto R, Higashiyama S, Mitamura T, Taniguchi N, Klagsbrun M, Mekada E (1994) Heparin-binding EGF-like growth factor, which acts as the diphteria toxin receptor, forms a complex with membrane protein DRAP27/CD9, which up-regulates functional receptors and diphteria toxin sensitivity. EMBO J 13: 2322–2330 8194524 10.1002/j.1460-2075.1994.tb06516.x PMC395097

[bib8] Iwamoto R, Mekada E (2000) Heparin-binding EGF-like growth factor: a juxtacrine growth factor. Cytokine Growth Factor Rev 11: 335–344 10959080 10.1016/s1359-6101(00)00013-7

[bib9] Jayne DG, Perry SL, Morrison E, Farmery SM, Guillon PJ (2000) Activated mesothelial cells produce heparin-binding growth factors: implications for tumor metastases. Br J Cancer 82: 1233–1238 10735511 10.1054/bjoc.1999.1068 PMC2363354

[bib10] Mills GB, May C, McGill M, Roifman CM, Mellers A (1998) A putative new growth factor in ascitic fluid from ovarian cancer patients: identification, characterization, and mechanism of action. Cancer Res 48: 1066–1071 3422589

[bib11] Mills GB, Moolenaar WH (2003) The emerging role of lysophosphatidic acid in cancer. Nat Rev Cancer 3: 582–591 12894246 10.1038/nrc1143

[bib12] Miyamoto S, Hirata M, Yamazaki A, Kageyama T, Hasuwa H, Mizushima H, Tanaka Y, Yagi H, Sonoda K, Kai M, Kanoh H, Nakano H, Mekada E (2004) Heparin-binding EGF-like growth factor and the LPA-induced ectodomain shedding pathway is a promising target for the therapy of ovarian cancer. Cancer Res 64: 5720–5727 15313912 10.1158/0008-5472.CAN-04-0811

[bib13] Morimoto H, Safrit JT, Bonavida J (1991) Synergistic effect of tumor necrosis factor-alpha- and diphteria toxin-mediated cytotoxicity in sensitive and resistant human ovarian tumor cell lines. J Immunol 147: 2609–2616 1918981

[bib14] Morishige K, Kurachi H, Amemiya K, Fujita Y, Yamamoto T, Miyake A (1991) Evidence for the involvement of transforming growth factor *α* and epidermal growth factor receptor autocrine growth mechanism in primary human ovarian cancers *in vitro*. Cancer Res 51: 5322–5328 1717146

[bib15] Normanno N, Bianco C, De Luca A, Maiello MR, Salomon DS (2003) Target-based agents against ErbB receptors and their ligands: a novel approach to cancer treatment. Endocr Relat Cancer 10: 1–21 12653668 10.1677/erc.0.0100001

[bib16] Penson RT, Shannon KE, Sharpless NE, Seiden MV (1998) Ovarian cancer an update on genetics and therpapy. Compr Ther 24: 477–487 9801846

[bib17] Prenzel N, Zwick E, Daub H, Leserer M, Abraham R, Wallasch C, Ullrich A (1999) EGF receptor transactivation by G-protein-coupled receptors requires metalloproteinase cleavage of proHB-EGF. Nature 402: 884–888 10622253 10.1038/47260

[bib18] Raab G, Klagsbrun M (1997) Heparin-binding EGF-like growth factor. Biochim Biophys Acta 1333: F179–F199 9426203 10.1016/s0304-419x(97)00024-3

[bib19] Salomon DS, Brandt R, Ciardiello F, Normanno M (1995) Epidermal growth factor-related peptides and their receptors in human malignancies. Crit Rev Oncol Hematol 19: 183–232 7612182 10.1016/1040-8428(94)00144-i

[bib20] Scambia G, Benedetti Panci P, Battaglia F, Ferrandina G, Baiocchi G, Greggi S, De Vincenzo R, Mancuso S (1992) Significance of epidermal growth factor receptor in advanced ovarian cancer. J Clin Oncol 10: 529–535 1548517 10.1200/JCO.1992.10.4.529

[bib21] Strachan L, Murison JG, Prestidge RL, Sleeman MA, Watson JD, Kumble KD (2001) Cloning and biological activity of epigen, a novel member of the epidermal growth factor superfamily. J Biol Chem 25: 18265–18271 10.1074/jbc.M006935200 11278323

[bib22] Tan MS, Tsai JC, Lee YJ, Chen HC, Shin SJ, Lai YH, Parrella MA, Bianchi C, Higashiyama S, Endege W, Lee ME, Tsai JH (1994) Induction of heparin-binding epidermal growth factor-like growth factor mRNA by protein kinase C activators. Kidney Int 46: 690–695 7996789 10.1038/ki.1994.322

[bib23] Umata T, Hirata M, Takahashi T, Ryu F, Shida S, Takahashi Y, Tsuneoka M, Miura Y, Masuda M, Horiguchi Y, Mekada E (2001) A dual signaling cascade that regulates the ectodomain shedding of heparin-binding epidermal growth factor-like growth factor. J Biol Chem 276: 30475–30482 11402047 10.1074/jbc.M103673200

[bib24] Westermann AM, Beijnen JH, Moolenaar WH, Rodenhuis S (1997) Growth factors in human ovarian cancer. Cancer Treat Rev 23: 113–131 9225962 10.1016/s0305-7372(97)90024-4

[bib25] Westermann AM, Havik E, Postma FR, Beijnen JH, Dalesio O, Moolenaar WH, Rodenhuis S (1998) Malignant effusion contain ; lysophosphatidic acid (LPA) –like activity. Ann Oncol 9: 437–442 9636836 10.1023/a:1008217129273

[bib26] Xiao YJ, Schwartz B, Washington M, Kennedy A, Webster K, Belinson J, Xu Y (2001) Electrospray lonization mass spectrometry analysis of lysophospholipids in human ascitic fluids: comparison of the lysophospholipid contents in malignant *vs* nonmalignant ascitic fluids. Anal Biochem 290: 302–313 11237333 10.1006/abio.2001.5000

[bib27] Xu Y, Gaudette DC, Boynton JD, Frankel A, Fang XJ, Sharma A, Hurteau J, Casey G, Goodbody A, Mellors A, Holub BJ, Mills GB (1995) Characterization of an ovarian cancer activating factor in ascites from ovarian cancer patients. Clin Cancer Res 1: 1223–1232 9815916

[bib28] Yeo EJ, Exton JH (1995) Stimulation of phospholipase D by epidermal growth factor requires protein kinase C activation in Swiss 3T3 cells. J Biol Chem 270: 3980–3988 7876145 10.1074/jbc.270.8.3980

